# Synthesis and characterization of PANI and PANI/nanometal oxides, photocatalytic and adsorbent applications

**DOI:** 10.55730/1300-0527.3542

**Published:** 2023-01-05

**Authors:** Bilge BUDAK, Sibel DEMİREL

**Affiliations:** Department of Chemistry, Faculty of Art, Kocaeli University Science, Kocaeli, Turkey

**Keywords:** PANI, nanocomposite, naproxen, UV, degradation

## Abstract

In this study, polymeric nanocomposites of PANI and PANI/nanometal oxides (Fe_2_O_3_, NiO, SnO_2_, WO_3,_ ZrO_2_) as photocatalysts were synthesized with a chemical polymerization method. Structural characterizations of synthesized polymeric nanocomposites were performed as follows: FT-IR, SEM, TEM and XRD, and TGA. The effect of pure nanometal oxides (Fe_2_O_3_, NiO, SnO_2_, WO_3,_ ZrO_2_) and polymeric nanocomposites on the degradation of naproxen, the drug active ingredient, was studied under visible light (UV-A at 365 nm) and in the absence of light. In this study, photocatalytic and adsorbent efficiency of polymeric nanometal oxides were investigated. In order to determine the effect of pure nanometal oxide particles and polymeric nanometal oxide composites on naproxen removal in light and dark environments, the percent removal with time was measured, and the band gap energies of each photocatalyst were examined using Tauc curves. Furthermore, the degradation reaction rate kinetic measurements of naproxen in light and dark conditions were carried out. From the experimental results, it was determined that pure nanometal oxides were not effective in light and dark environments, but the synthesized PANI nanometal composites were effective in the removal of naproxen in wastewater in both light and dark environments.

## 1. Introduction

Water pollution has become a serious problem worldwide due to increasing agricultural and industrial activities because of the increase in the world population. Due to the pollution caused by hazardous wastes, the quality of river and groundwater has begun to deteriorate to a large extent, and these toxic pollutants have adversely affected human health and the quality of the ecosystem [1–3]. Major sources of water pollution include heavy metal ions, dyes, pharmaceuticals, pesticides, phenols, polychlorinated biphenyls, haloacetic acids, disinfection by-products, and other synthetic chemicals [4–9]. Pharmaceutical active substances, which are among the main sources of pollution in wastewater, are biologically active substances and are freely soluble in water. However, some active pharmaceutical ingredients are not stable in aqueous media. Therefore, these compounds are very difficult to degrade in the wastewater environment [1–4].

Naproxen, one of the drug’s active ingredients, is a nonsteroidal antiinflammatory drug with analgesic and antipyretic properties, used as a pain reliever and for the treatment of rheumatoid arthritis [5]. Naproxen has been detected at high concentrations at very different amounts in surface waters, groundwaters, and drinking waters ranging from 10 ng L^−1^ to 10 μg L^−1^ [6–8]. Few studies have been found on the photocatalytic removal of naproxen using polymeric composites [6–11]. When the studies were reviewed, it was determined that studies with advanced oxidation methods provided an advantage in the removal of drug residues.

Photochemistry (photocatalytic degradation-decomposition), which is a new field of study and included in advanced oxidation techniques, aims to remove organic impurities. Advanced oxidation processes (AOP) provide removal of organic impurities through the production of reactive oxidants/radicals such as OH and O_2_^−^ under different light sources (UV, visible, microwave, and sunlight). It has been determined that advanced oxidation processes are superior to conventional treatment processes (adsorption, membrane filtration, osmosis, ion exchange, and chemical precipitation, etc.) for the complete decomposition of persistent organic impurities [11–23]. In the literature, inorganic metal oxides such as V_2_O_5_, MnO_2_, WO_3_, Nb_2_O_5_, SnO_2_, TiO_2_, ZnO, Fe_2_O_3_, and Fe_3_O_4_ are used as catalysts in water treatment processes [24–28]. The low cost and environmental friendliness of these heterogeneous semiconductor photocatalysis are the reasons why they are preferred for wastewater treatment [29–34]. However, these metal oxides, besides their advantages such as faster electron-gap recombination and the presence of wide band gap [35–38], also have disadvantages as they have low quantum efficiency and can only be activated by ultraviolet radiation [22, 23].

Metal oxide doping is applied to the semiconductor polymer to minimize the disadvantages of semiconductor nanometal oxides and create a visible light activated photocatalyst with high photocatalytic efficiency and stability. For this purpose, recently, polyaniline (PANI) has been one of the preferred conductive polymers. There are many reasons why polyaniline is an important polymer; its semiconductor, flexibility, stability, wide pH range, redox activity, and conductivity make it an important polymer type. By doping semiconductor metal oxides (such as TiO_2_, ZnO, ZrO_2_, etc.) to semiconductor polymers such as PANI, the band gap energy decreases in the visible region, and the photocatalytic effect is achieved by facilitating electron transition from the conduction band to the valence band [39–42]. In other words, although these polymeric nanocomposites increase, the photocatalytic efficiency in the visible region, it has been determined that these composites are extremely effective as an adsorbent in the studies [43].

In this study, pure nanometal oxides (Fe_2_O_3_, NiO, SnO_2_, WO_3,_ and ZrO_2_) and synthesized PANI/(Fe_2_O_3_, NiO, SnO_2_, WO_3,_ and ZrO_2_) nanometals were used in the removal of naproxen, which is used as an active drug in wastewater. The photocatalytic efficiency of the composites under UV-A irradiation and the adsorbent activity in the absence of light was investigated. The contribution of band gap energies to the photocatalytic removal of naproxen was determined by constructing Tauc curves. In addition, the degradation reaction kinetics of naproxen was investigated by trying different kinetic models.

## 2. Experimental

### 2.1. Chemicals

Iron oxide (Fe_2_O_3_, CAS: 1309-37-1, Mw: 159. 68 g/mol), nickel oxide (NiO, CAS: 1313-99-1, Mw: 74.69 g/mol), tin dioxide (SnO_2_, CAS: 18282-10-5, Mw: 150.71 g/mol), tungsten trioxide (WO_3_, CAS: 1314-35-8, Mw: 231.84 g/mol) and zirconium dioxide (ZrO_2_, CAS: 1314-23-4, Mw: 123.22 g/mol) pure nanometal oxides, aniline (ANI, C_6_H_7_N, 99.5% purity), ammoniumperoxydisulfate (APS, (NH_4_)_2_S_2_0_8_), ethanol (C_2_H_6_O), hydrochloric acid (HCl), naproxen (NPX), CAS: 26159-34-2, C_14_H_13_NaO_3_) was obtained from Sigma Company. All chemicals used for the synthesis of nanomaterials were used as received without any purification.

### 2.2. Synthesis of PANI and PANI/metal oxides (Fe_2_O_3_, NiO, SnO_2_, WO_3_, and ZrO_2_)

Aniline solution (1 mL) was added dropwise to the previously prepared 2 M 70 mL of HCl solution. Next, it was mixed at constant speed on a magnetic stirrer for 1 h. APS solution prepared by dissolving 2.5 g of APS in 20 mL of deionized water was added dropwise to the solution, which was mixed at a constant rate, and it was allowed to polymerize at room temperature for 5 h. The product obtained as a result of polymerization was washed with HCl, ethyl alcohol, and deionized water and filtered under vacuum. The resulting solid product was dried in an oven at 60 °C [24]. Synthesis of polymeric nanocomposites of PANI/metal oxide (PANI/Fe_2_O_3_, PANI/NiO, PANI/SnO_2_, PANI/WO_3_, and PANI/ZrO_2_) was performed using a procedure similar to PANI synthesis. Metal oxide nanoparticles (0.5 g) were added to the HCl solution and allowed to mix in an ultrasonic bath for 1 h and in a magnetic stirrer for 1 h. While mixing continued, the above procedures were repeated by adding 1 mL of aniline to the solution [29, 39].

### 2.3. Characterization

Structural characterizations of nanocomposites used as photocatalysts were determined by FT-IR (Perkin Elmer-2000 infrared spectrometer in the wavelength range of 4000–400 cm^−1^), SEM (Cressington 208 C device), XRD (Rigaku Ultima-IV X-ray diffraction device) and TEM (JEOL-2100 LaB6) and thermal analysis using TGA (10 °C/min. heating rate with Mettler Toledo analyzers). In addition, band gap energies and photocatalytic activity measurements were determined by using UV-spectroscopy (Perkin Elmer UV Lambda 25 spectrophotometer).

### 2.4. Photocatalytic activity measurements

After determining the maximum wavelength of naproxen as 228 nm using a UV spectrophotometer, the calibration curve was drawn. Then, the photocatalytic removal trials of naproxen were performed under UV A (400–320) irradiation using a UV cabinet at 365 nm. A high-pressure sodium lamp (OSRAM, VIALOX SON-T, 400 W) that emitted light mainly between 400 and 700 nm wavelengths was used in the tests. The degradation process was carried out by adding 0.5 g of catalyst to the naproxen solution prepared at a certain concentration (50 mg/L) and mixing it in the magnetic stirrer in the UV cabinet. The same process was repeated in the absence of light. Concentration and wavelength measurements were made with a spectrophotometer by taking samples from the solution at certain time intervals. All experimental studies were carried out at room temperature. The photocatalytic degradation experiments of the drug continued under UV-A irradiation and in the absence of light at certain time intervals until the equilibrium concentration was reached.

The percent removal of naproxen from the results obtained after photocatalytic treatments was calculated from the following equation [45].


(1) $$C\%=\left(\frac{\left(C_{0}-Ce\right)}{C_{0}}\right)\times 100.$$

C_0_ is the initial concentration of the active ingredient (mg/L); C_e,_ is the equilibrium concentration of the active ingredient (mg/L); C % is the percent removal amount.

### 2.5. Band gap energy determination

The way to examine the optical absorption spectrum of a material is to determine its band gap energies. The band gap energy can be determined using the Tauc curve. Accurate determination of the band gap energy is very important in estimating the photophysical and photochemical properties of semiconductors. In the Tauc method, the energy-dependent absorption coefficient α can be explained by the following equation:


(2) $${\left(\alpha \cdot \text{h}\nu \right)}^{1/\gamma }=\;\text{B }(\text{h}\nu -{\text{E}}_{\text{g}}),$$

where h is Planck’s constant, ν is the frequency of the photon, E_g_ is the band gap energy, and B is a constant. The γ factor depends on the nature of the electron transition and is equal to 1/2 or 2 for the direct and indirect pass band gaps, respectively.

According to the theory of P. Kubelka and F. Munk presented in 1931 [33], the measured reflection spectra can be converted into corresponding absorption spectra by applying the Kubelka-Munk function. Using the Kubelka Munk function and Tauc curves, (α·hν)^2^ vs. energy (eV) graph is drawn, and the band gap energy value is calculated from the backward extrapolation of the graph [32–35].

## 3. Results and discussion

### 3.1. Characterization results

#### 3.1.1. FT-IR results

**Figure 1:**
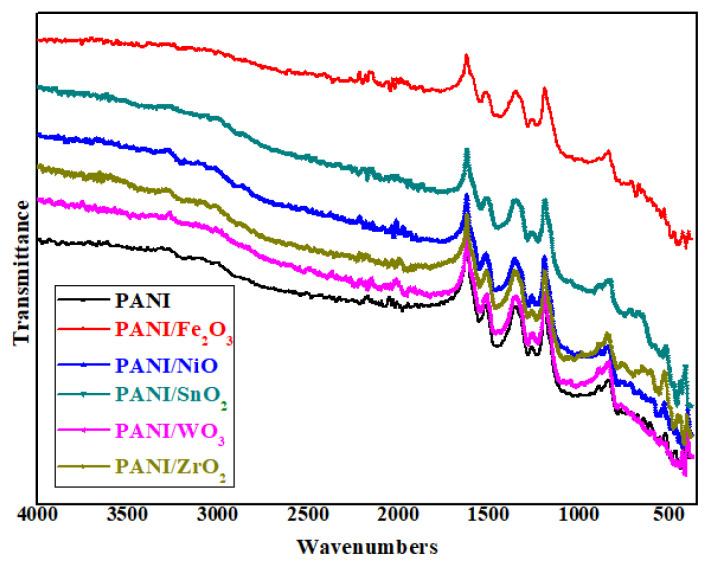
FT-IR spectrum curves for polymeric nanocomposites of PANI, PANI/Fe_2_O_3_, PANI/NiO, PANI/SnO_2_, PANI/WO_3_, PANI/ZrO_2_.

The FT-IR spectrum curves of polymeric nanocomposites are presented in Figure 1. The characteristic absorption peaks of the composites are given in Table 1.

For PANI, C=C tensile vibration of the quinoid ring at 1553 cm^−1^, C=C tensile vibration of the benzenoid ring at 1449 cm^−1^, C-N tensile vibration at 1282 cm^−1^, C-H in-plane deformation and C-H out-of-plane deformation are observed at wavelengths of 1121 cm^−1^, 817 cm^−1^, respectively (Figure 1). In addition, the characteristic peak of N-H stretching mode is observed for PANI at 3264 cm^−1^ [32, 38]. When we look at the FT-IR spectrum of the PANI/Fe_2_O_3_ polymeric nanocomposite, there are main characteristic peaks attributed to Fe_2_O_3_ at 447.87 cm^−1^, and 546.6 cm^−1^, although there are some shifts relative to PANI. This indicates that Fe_2_O_3_ nanoparticles enter the PANI polymeric network structure [39]. The FT-IR spectrum of PANI/NiO is almost similar to that of PANI, but some peaks are slightly shifted (Table 1) [40]. In addition, due to NiO nanoparticles in the polymeric network structure, the peaks observed at 600 cm^−1^ and 800 cm^−1^ were related to the Ni–O stretching vibration mode [40]. In the spectrum of PANI/SnO_2_, there are shifts in the characteristic peaks of PANI, as seen in Table 1. The shift corresponds to the O–Sn bond and the free oxy hydroxide [41]. In the FT-IR spectrum of PANI/WO_3_, there are small shifts compared to PANI, which, as in other composites (Table 1), can be explained by the attributed band [40]. As seen in Table 1, in the FT-IR spectrum of PANI/ZrO_2_, there are shifts in the characteristic peaks of PANI, and the characteristic peak related to the Zr-O stretching, indicating that the presence of ZrO_2_ nanometal oxide is observed at 500–600 cm^−1^ [38].

As seen in Table 1, the FT-IR spectrum peaks of all polymeric composites were similar to the characteristic peaks of pure PANI but caused a slight shift due to the nanometal oxides added to the PANI structure. Such shifts show that nanometal oxides participate in the polymeric network structure. This result is also supported by SEM and TEM images.

#### 3.1.2. FE-SEM and HR-TEM results

**Figure 2:**
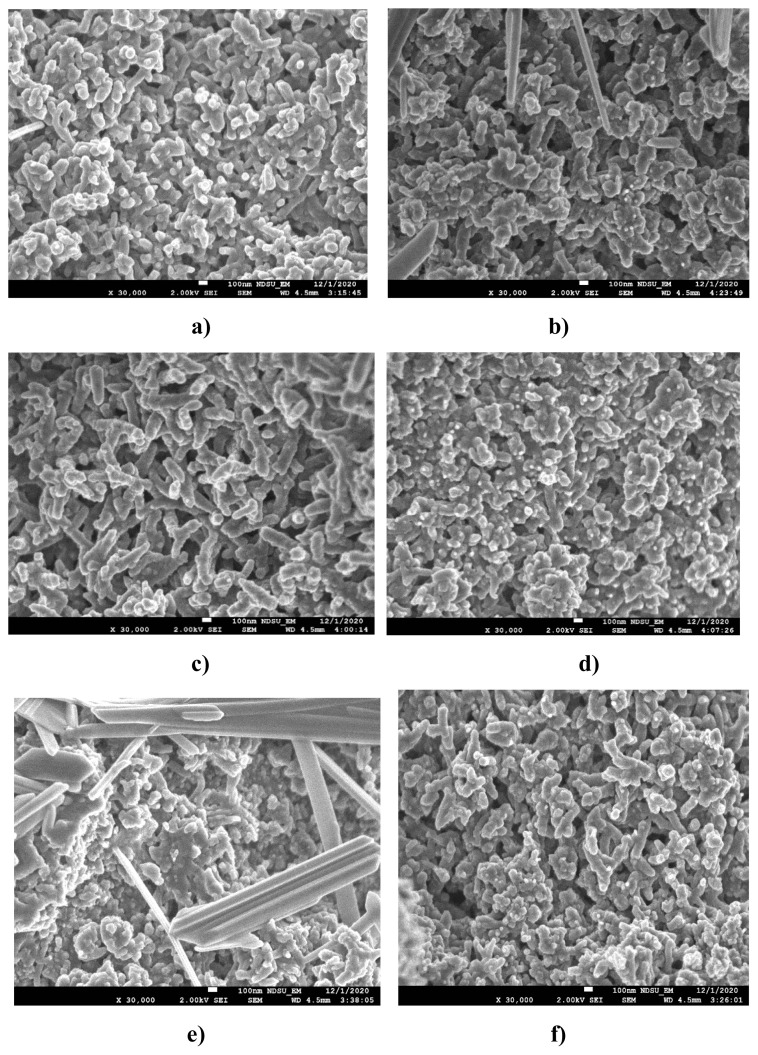
SEM images **a)** PANI, **b)** PANI/Fe_2_O_3,_ **c)** PANI/NiO, **d)** PANI/SnO_2,_ **e)**PANI/WO_3_, **f)** PANI/ZrO_2._

**Figure 3:**
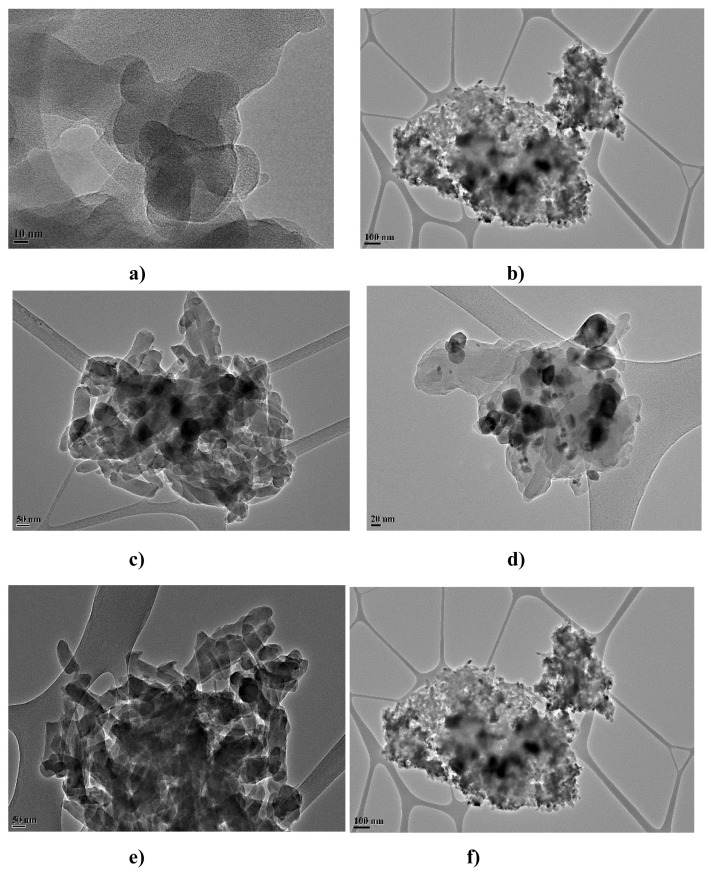
TEM images **a)** PANI, **b)** PANI/Fe_2_O_3_, **c)** PANI/NiO, **d)** PANI/SnO_2_, **e)**PANI/WO_3_, **f)** PANI/ZrO_2_.

The morphology of PANI and PANI/metal oxide nanocomposites with and without nanometal oxide is presented in Figures 2a–2f as SEM images. In Figure 2a, it is seen that PANI has a spherical structure and shows a homogeneous distribution. In Figure 2b, it is understood that Fe_2_O_3_ nanoparticles, which are rod-shaped in the SEM image of PANI/Fe_2_O_3_, are located in the polymeric network structure. In Figure 2c, it is seen that crystalline NiO nanoparticles enter the polymeric network structure, while in Figure 2d, crystalline SnO_2_ nanoparticles are located in the polymeric network structure. The agglomerated structure of WO_3_ nanometal oxide in rod form in the PANI polymeric network is shown in Figure 3e [38]. With the addition of ZrO_2_ to the polymeric composite, it is seen that metal oxide particles enter the composite structure, nanoparticles come together in some regions (Figure 2 f), and ZrO_2_ nanoparticles form a spiral structure on the polymeric surface [29].

**Figure 4:**
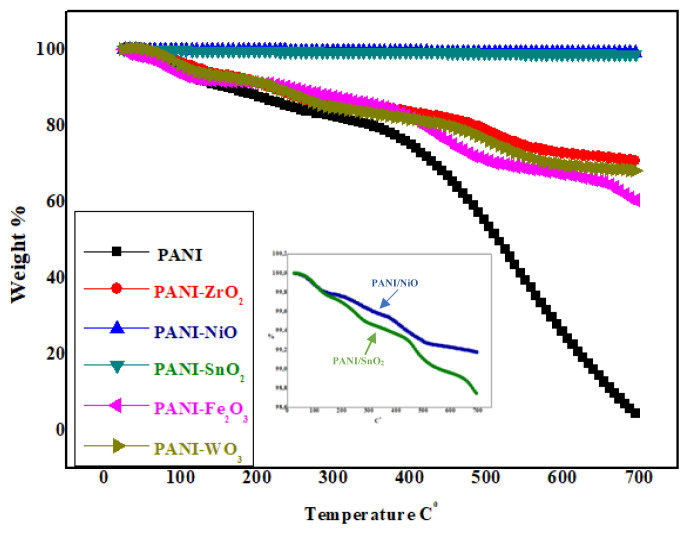
TGA curves of polymeric nanocomposites (PANI, PANI/Fe_2_O_3_, PANI/NiO, PANI/SnO_2_, PANI/WO_3,_ PANI/ZrO_2_) (heating rate 10 °C min^−1^).

TEM images of PANI and PANI/nanometal oxide composites are given in Figures 3a–3f. Figure 3a shows the cloudy network structure of PANI molecules [29]. Here, they appear as small, spherical but closely packed particles, possibly due to π–π* interactions between PANI molecules [36]. In the TEM image in Figure 4b, the PANI/Fe_2_O_3_ nanoparticles are uniform and monodisperse, and the Fe_2_O_3_ layers are located in a polymeric network structure without disturbing the spherical particle shape [46]. The crystalline spherical structure of NiO naoparticles in PANI is shown in Figure 3c [44]. In Figure 3d, SnO_2_ nanoparticles in crystal tetragonal rutile with a polymeric network structure are located in the polymeric structure [45]. The rod-like structure of WO_3_ nanoparticles within the PANI polymeric network is seen in Figure 3e [43]. ZrO_2_ distribution in PANI shows that ZrO_2_ particles cluster in the polymeric network structure in the cloud in PANI, and ZrO_2_ nanoparticles are abundantly located in the polymeric network structure and agglomerate in some regions (Figure 3f) [42]. TEM images determined that nanometal oxide particles in the polymeric composite entered the polymeric network structure and agglomerated in some regions.

#### 3.1.3. TGA results

TGA results of nanocomposites are shown in Figure 4. Thermogravimetric analysis was performed in a dynamic nitrogen atmosphere (30 cm^3^ min^−1^) using a Mettler Toledo device with a heating rate of 10 °C min^−1^ in the temperature range of 25–700 °C, using micro- and ultramicrobalances. In the PANI, mass loss up to 105 °C is due to moisture, but weight loss due to absorbed moisture and solvent is observed at heating up to 230 °C. The weight loss after 400 °C also shows that the material can remain intact until that temperature [46]. The mass loss in PANI polymeric composite was around 98%–99%.

In other composites with all metal oxide additives, thermal reduction occurs in three stages. The first stage is the removal of water, the second stage reduction is due to the release of anions, and the third stage is due to the decomposition of the polymer. Interdecay steps show that the interdegradation products are stable at certain temperature ranges [47–53].

In PANI/Fe_2_O_3_, the mass loss was 40%, and 60% of the material remained intact. Mass loss in PANI/WO_3_ was around 35%. In PANI/ZrO_2_, the mass loss was around 30%, and 70% of the material remained intact. Mass loss in PANI/NiO and PANI/SnO_2_ nanocomposites is negligible and was determined as 1% and 2%, respectively. The low amount of mass loss indicates that the thermal endurance of the samples is high. Accordingly, nanometal oxides added to PANI increased the thermal strength of polymeric composites, and the highest thermal strength was observed in PANI/NiO and PANI/SnO_2_ composites [54].

#### 3.1.4. XRD results

**Figure 5:**
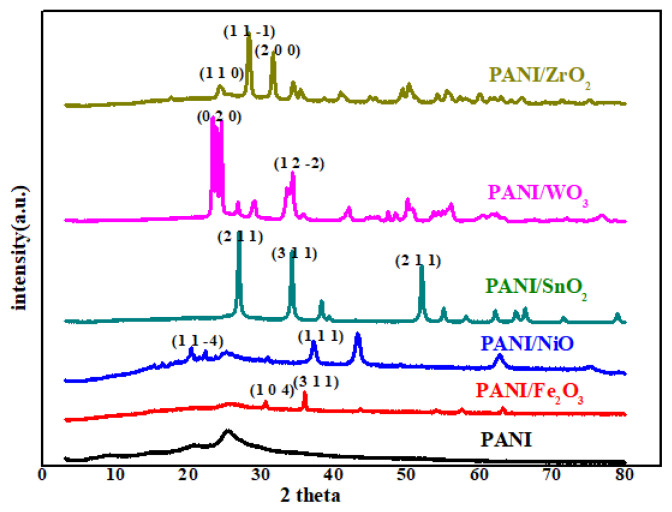
XRD image of PANI and PANI/metal oxide polymeric nanocomposites (PANI, PANI/Fe_2_O_3_, PANI/NiO, PANI/SnO_2_, PANI/WO_3,_ PANI/ZrO_2_).

XRD models of nanoparticles synthesized by the chemical polymerization method are showed in Figure 5. For PANI powders, there are four characteristic peaks at an angle of 2θ. These are 14.786°, 20.022°, 24.861°, 35.434°. In the literature, 2θ degrees for pure PANI polymer are 20.30° and 25.10°. In general, polymers can be expected to be amorphous; the synthesized PANI polymers are crystalline due to the structural nature of the benzenoid and quinoid functional groups.

The weak diffraction peaks obtained in the PANI/Fe_2_O_3_ composite are at 2θ at 24.33°, 33.28°, 35.77°, 40.92°, 49.56°, 54.17°, 62.55°, and 64.10°, and the Fe_2_O_3_ in the structure (JCPDS card No. 33-0664) was observed to be consistent with the standard rutile phase, except for some peaks (43.64° and 63.201°) [55]. This is an indication that Fe_2_O_3_ is doped into the polymer matrix. After NiO was incorporated into the PANI matrix to form the PANI/NiO composite, it was observed at 2θ = 14° (weak), 20°, 25°,37°, 43°, and 62°, respectively. This diffraction pattern corresponds to cubic NiO and is compatible with standard JCPDS values (JCPDS: 47-1049) [56]. XRD patterns for PANI/SnO_2_ nanocomposite show distinct orientation peaks at 26.5°, 38.28°, and 52°, corresponding to (110), (101), and (211) planes of tetragonal SnO_2_, respectively (JCPDS: 41-1445) [57,58].

In the XRD peak of the PANI/WO_3_ polymeric nanocomposite, the potential peak, caused by the interactions of the polyaniline and WO_3_ particles, indicating the crystal structure change of the polyaniline matrix, was detected at 33.50°. The peak intensity due to this crystal structure change is due to the morphology of the nanocomposite, and peaks of 23.80°, 24.08°, and 26.92° show strong agreement with those in the standard XRD data card (JCPDS # 43-1035) as well as their corresponding values [59,60]. The characteristic peaks at the 2θ angle of the PANI/ZrO_2_ nanocomposite are as follows: 17.50°, 24.03°, 35.34°, 44.79°, and 50.124°. In the literature, the degrees of 2θ angles for the ZrO_2_ semiconductor metal oxide particle are 24.20°, 28.2°, 31.40°, and 34.30°. In the XRD models of PANI/ZrO_2_ nanoparticles, there is a significant shift in the peaks of nanocomposites prepared by adding ZrO_2_ [29]. It can be seen that all the diffraction peaks determined in the XRD model correspond to the monoclinic crystalline phase (Baddeleyite-JCPDS 65-1025) [61].

In the XRD models of nanoparticles, there are shifts in the peaks of nanocomposites prepared by adding metal oxides compared to PANI, and the XRD results obtained are compatible with the literature data.

### 3.2. Photocatalytic activity results

**Figure 6:**
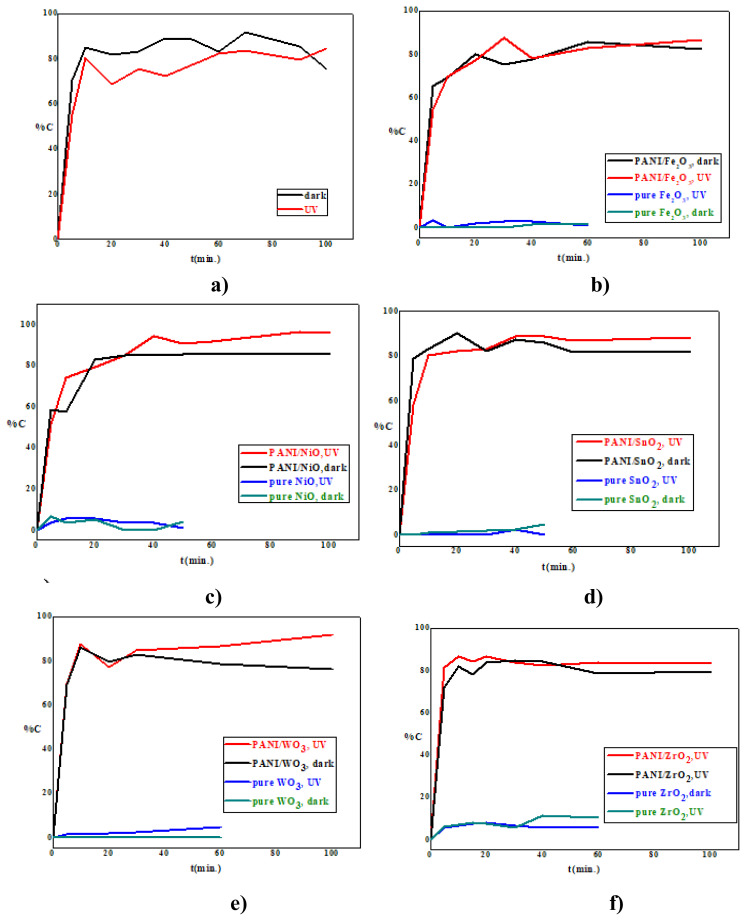
Naproxen removal versus time (C%-t) curves **a)** PANI, **b)** PANI/Fe_2_O_3_, **c)** PANI/NiO, **d)** PANI/SnO_2_, **e)** PANI/WO_3,_ **f)** PANI/ZrO_2._

For pure nanometal oxides (Fe_2_O_3_, NiO, SnO_2_, WO_3_, and ZrO_2_) and polymeric composites with and without nanometal oxides (PANI, PANI/Fe_2_O_3_, PANI/NiO, PANI/SnO_2_, PANI/WO_3_, and PANI/ZrO_2_), the variation of the percent removal of naproxen with time under UV-A irradiation and in the absence of light is given in Figures 6a–6f. As can be seen from the curves, it was determined that pure nanometal oxides were not effective enough in naproxen removal under UV-A irradiation and in the absence of light for 60 min.

As a result of the use of PANI as a photocatalyst, approximately 80% removal was achieved in 100 min under UV irradiation, while in the same period without light, nearly 80% removal was achieved (Figure 6a). PANI/Fe_2_O_3_ polymeric nanocomposite also provided 80% removal in 100 min under UV irradiation and in the dark. In this composite, the removal of naproxen was the same in light and in the absence of light. In the removal of naproxen, the adsorbent efficiency of the composite is higher than the photocatalytic activity (Figure 6b).

On the other hand, PANI/NiO nanocomposite provided 98% removal in 100 min under UV-A irradiation and 85% removal in the absence of light. In the removal of naproxen, the removal of PANI/NiO nanocomposite increased in the same period under UV-A irradiation with the effect of irradiation. In other words, although the photocatalytic efficiency of the PANI/NiO composite was higher in naproxen removal, it was determined that it was effective in its adsorbent property (Figure 6c).

PANI/SnO_2_ composite achieved 90% naproxen removal under UV irradiation, and nearly 80% removal in the same time interval in the absence of light (Figure 6d). The PANI/WO_3_ polymeric nanocomposite also provided 90% removal in 100 min but approximately 78% removal in the absence of light (Figure 6e). PANI/ZrO_2_ composite provided 82% naproxen removal under UV irradiation and 78% naproxen removal in the absence of light (Figure 6f)

From the photocatalytic results obtained, it was determined that the pure semiconductor nanometal oxides were not effective in removing naproxen under UV-A irradiation and in the absence of light. (Figures 6b–6f). It has been observed that polymeric composites are effective in naproxen removal in both light and nonlight conditions. The order of photocatalytic activity polymeric composites is: PANI/NiO > PANI/WO_3_ >PANI/SnO_2_ >PANI/ZrO_2_ >PANI/Fe_2_O_3_, and it has been observed that composites are effective in naproxen removal in both light and nonlight conditions. In addition, it is seen that the efficiency of naproxen removal of polymeric composites is high in the absence of light. This shows that the adsorbent properties of the photocatalysts used are effective in the absence of light. Therefore, it was determined that the polymeric composites used were effective in the removal of naproxen in with and without light conditions. From the results obtained, it was determined that NiO nanoparticles coated with PANI chains were the most effective in removing naproxen in photocatalytic experiments (PANI/NiO). This result is also supported by band gap energy changes. Thus, the most effective PANI/NiO nanocomposite was the most effective polymeric nanocomposite, providing 96% degradation efficiency under UV-A irradiation and 85% degradation activityin the dark.

### 3. 3. Band gap energy results

**Figure 7:**
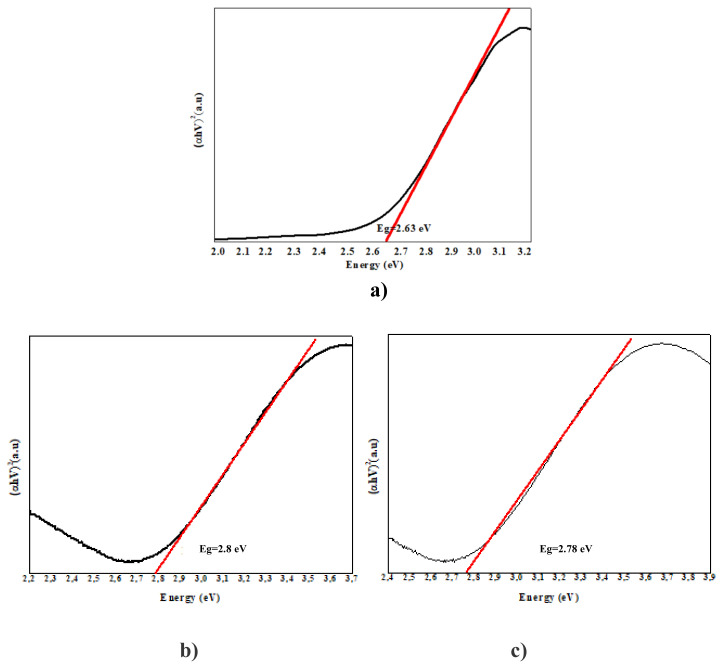
Tauc curves **a)** Fe_2_O_3_, **b)** PANI/Fe_2_O_3_.

**Figure 8:**
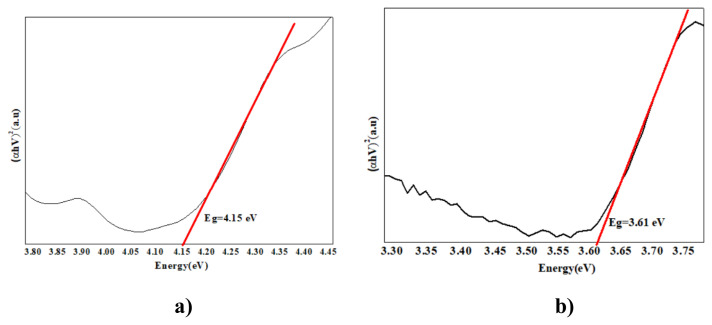
Tauc curves **a)** NiO, **b)** PANI/NiO.

**Figure 9:**
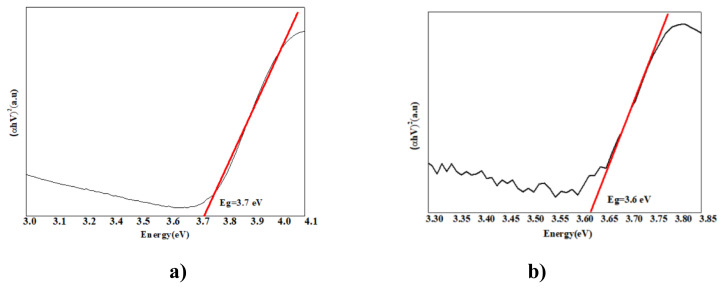
Tauc curves **a)** SnO_2_, **b)** PANI/SnO_2_.

**Figure 10:**
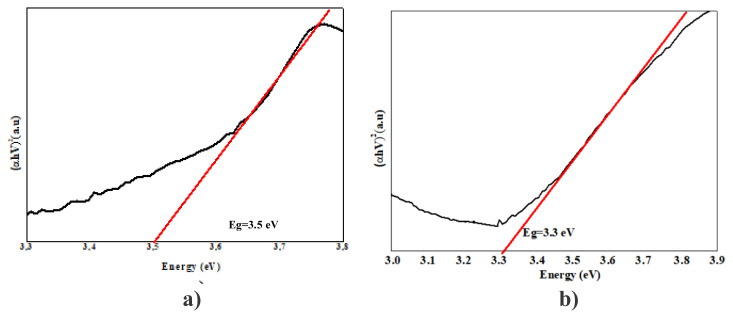
Tauc curves **a)** WO_3_ **b)** PANI/WO_3_.

**Figure 11:**
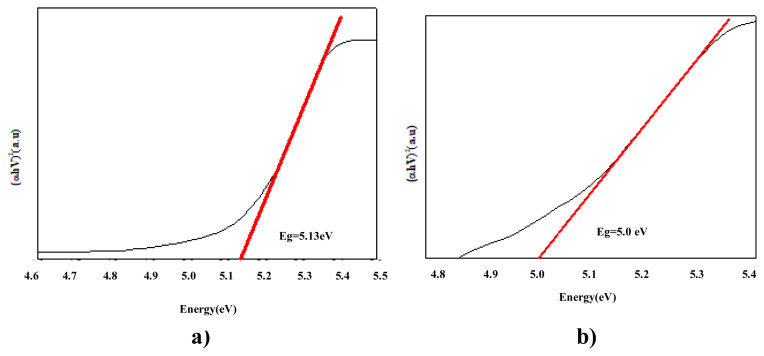
Tauc curves **a)** ZrO_2_, **b)** PANI/ZrO_2_.

In our study, band gap energy changes in photocatalytic removals were investigated using the Kubelka Munck function and the Tauc curve. Band gap values were calculated from the plot of (αhν)_2_ vs. hν using the curves given in Figures 7–11.

As PANI shows a better result, the PANI band gap energy is shown in Figure 7a. PANI was dissolved in solution and the maximum wavelength was measured around 470 nm and band gap energy was measured 2.6 e V using a spectrophotometer. For pure nano Fe_2_O_3_, the maximum wavelength was measured around 442 nm using a spectrophotometer, and the band gap energy was determined as 2.8 eV [69]. The wavelength of the PANI/Fe_2_O_3_ polymeric nanocomposite was 445 nm, and the band gap energy was 2.78 eV (Figure 7). The maximum wavelength of NiO nanoparticles was 298 nm [70]. The band gap energy was also determined as 4.15 eV (Figure 8a) [71]. The wavelength of the PANI/NiO polymeric nanocomposite was 341 nm, and the band gap energy decreased to 3.63 eV (Figure 8b). Here, the band gap energy decrease indicates that the electron transition from the valence band to the conduction band is facilitated.The maximum wavelength of SnO_2_ was measured around 335 nm using a spectrophotometer, and the band gap energy was determined as 3.7 eV (Figure 9a) [72]. The wavelength of the PANI/SnO_2_ polymeric nanocomposite was 343nm and the band gap energy was determined to be 3.61 eV (Figure 9b). The maximum wavelength of WO_3_ was 354 nm, the band gap energy is 3.5 eV (Figure 10a) [73] the wavelength of the PANI/WO_3_ polymeric nanocomposite was 375 nm, and the band gap energy was determined to be 3.3 eV (Figure 10b). The maximum wavelength of ZrO_2_ nanoparticles was 242 nm, and band gap energy was 5.13 eV (Figure 11a) [74]. PANI/ZrO_2_ wavelength was 248 nm, and band gap energy was determined as 5.0 eV (Figure 11b).

Calculated band gap energies of metal oxides alone are higher than the value of the semiconductor doped with the addition of nanometal oxides. The band gap energies of the polymeric nanocomposites decreased compared to the pure nanometal oxide. A shift to higher wavelengths was observed, corresponding to a decrease in band gap energies and hence higher absorbance of visible light. With the decrease of the band gap energies of the polymeric nanometal oxides, the transition of electrons from the valence band to the holes in the conduction band is facilitated, increasing the photocatalytic efficiency in the visible region [75].

### 3.4. Photocatalytic degradation mechanism

**Figure 12:**
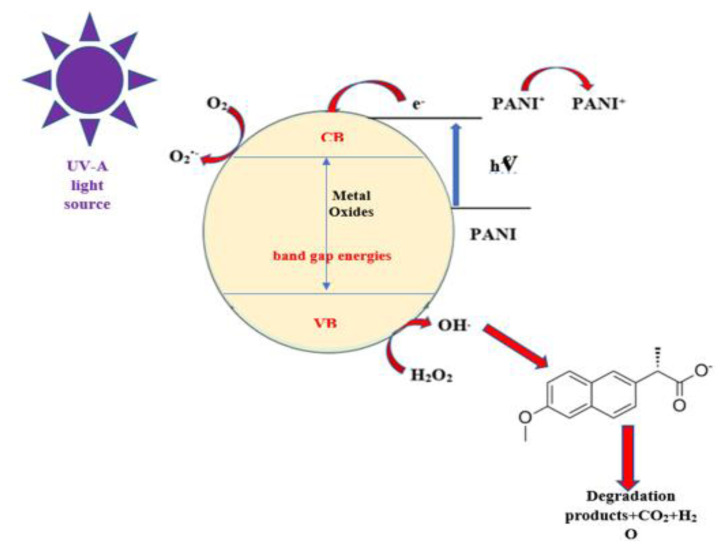
Photocatalytic reaction mechanism of naproxen removal.

In polymeric nanocomposites, the band gap energy decreases, and the transition of electrons from the conduction band to the valence band becomes easier, thus increasing the photocatalytic efficiency. The mechanism of the photocatalytic reaction of PANI and PANI/Metal oxide (Fe_2_O_3_, NiO, SnO_2_, WO_3_, and ZrO_2_) polymeric nanocomposite in the removal of naproxen drug active substance used as a pollutant in wastewater in visible (UV-A) light is shown schematically in Figure 12.

The advanced photocatalytic activity mechanism is based on the synergistic effect between PANI and metal oxide nanoparticles (Fe_2_O_3_, NiO, SnO_2_, WO_3_, and ZrO_2_). The photocatalytic degradation reaction steps occurring on the polymeric composite surface are as follows [62–66]:


(3) $$\text{PNAI}/(\text{MOx})+\text{hv}\to \text{PANI}+(\text{MOx})\;\text{ya da }(\text{MOx})+{\text{e}}_{\text{CB}}$$

(4) $${\text{e}}_{\text{CB}}+{\text{O}}_{2}\to {\text{O}}_{2^{•-}}$$

(5) $$\text{PANI}+(\text{MOx})\to \text{PANI}+(\text{MOx})+{{\text{hv}}^{+}}_{\text{VB}}$$

(6) $${{\text{hv}}^{+}}_{\text{VB}}+({\text{H}}_{2}\text{O }{\text{H}}^{+}+{\text{OH}}^{-})\to {\text{H}}^{+}+{\;}^{•}\text{OH}$$

(7) $$\text{Naproxen}+{\;}^{•}\text{OH}\to {\text{CO}}_{2}+{\text{H}}_{2}\text{O}+\text{degradation products}$$

PANI and metal oxide nanoparticles (Fe_2_O_3_, NiO, SnO_2_, WO_3_, and ZrO_2_), when irradiated with UV light, generate electron-hole pairs that can react with water to form hydroxyl and superoxide radicals. These radicals oxidize naproxen molecules.

However, depending on the band gap of the nanometal oxide, electron-hole pairs occur. Free electrons react with O_2_ to produce the superoxide radical (·O_2_^−^), and holes (h^+^) react with OH and H_2_O to produce a hydroxyl radical (·OH). The hydroxyl radicals that are formed attach to naproxen and cause the formation of CO_2_, H_2_O, and degradation products [67,68].

### 3.5. Kinetic studies

Pseudofirst-order [76], pseudosecond-order [77], and intraparticle diffusion models [78] were used to examine the reaction kinetics and control mechanism and to interpret the experimental data. For all examined systems, the appropriate pseudosecond-order chemical reaction kinetics in naproxen removal was determined by the correlation coefficient.

Pseudosecond-order kinetic model of Blanchard et al. (1984) was stated as follows:


(8) $$\frac{dqt}{dt}=k_{2}{\left(q_{e}-q_{t}\right)}^{2}.$$

Later, this equation was rearranged in linear format by Ho (1995) and it is as follows:


(9) $$\frac{t}{qt}=\frac{t}{qe}+\frac{1}{k_{2}\cdot q_{e}^{2}}.$$

Here, q_e_ is the amount of substance adsorbed per gram of adsorbent at equilibrium (mg/g), q_t_ is the amount of substance adsorbed per gram of adsorbent at any time (mg/g), k_2_ is the rate constant (g/(mg.min)). The initial adsorption rate is h = k_2_.q_e_^2^. The rate constant k_2_ and theoretical q_e_ values are calculated from the intersection and slope of the graph of t/against t, respectively.

**Figure 13:**
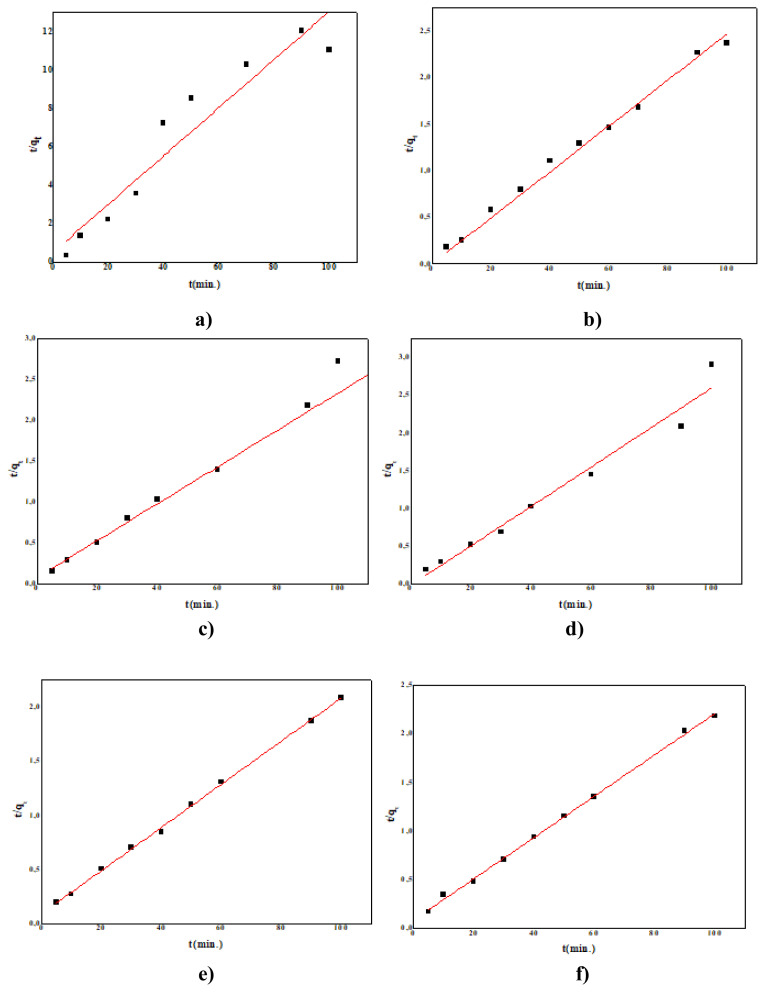
t/qt-t graphs of pseudo 2nd order kinetic model with and without light **a)** PANI in light, **b)** PANI in dark, **c)** PANI/Fe_2_O_3_ under UV irradiation, **d)** PANI/Fe_2_O_3_ in dark, **e)** PANI/NiO under UV irradiation, **f)** PANI/NiO in dark, **g)** PANI/SnO_2_ under UV irradiation, **h)** PANI/SnO_2_ in dark, **i)** PANI/WO_3_ under UV irradiation, **j)** PANI/WO_3_ in dark, **k)** PANI/ZrO_2_ under UV irradiation, **l)** PANI/ZrO_2_ in dark.

In our study, the kinetic constants calculated from the graphs in Figure 13 obtained with the pseudosecond-order rate equations in the removal of naproxen in light and dark conditions are given in Table 2. For all the systems studied, it was determined that the experimental data of second-order chemical reaction kinetics provided the best correlation. Although the data of the proposed pseudofirst-order model and intramolecular diffusion model are compatible for some reaction periods, the pseudosecond-order model provides the best correlation for all examined systems [79]. It is understood that PANI/NiO has the best effect as a photocatalyst and adsorbent, with a high reaction rate constant. In addition, the reaction rate constants in the light environment were higher than in the nonlight environment, and UV-A irradiation increased the degradation reaction of naproxen (Table 2).

## 4. Conclusion

PANI and PANI/Metal oxide (Fe_2_O_3_, NiO, SnO_2_, WO_3_, and ZrO_2_) polymeric nanocomposites were successfully synthesized with the chemical polymerization method. The structural and thermal properties of the synthesized polymeric nanocomposites were determined by; FT-IR, SEM, TEM, XRD, and TGA. According to the characterization results, it was determined that there is a strong interaction between semiconductor polymers and metal oxides. To determine the photocatalytic efficiency of pure nanometal oxides (Fe_2_O_3_, NiO, SnO_2_, WO_3_, and ZrO_2_), naproxen removal with pure nanometals without PANI/nanometal oxide semiconductor modification was tried, and it was determined. From the results, it is seen that pure nanometal oxides were ineffective in naproxen degradation. PANI/NiO nanocomposite was determined to be the most effective polymeric nanocomposite since it has a photocatalytic degradation efficiency of 96% under UV-A irradiation and 85% effective in the absence of light. It has been observed that polymeric nanometal oxides are photocatalytically effective in naproxen removal and in adsorption activities from experimental studies conducted in the absence of light. Band gap energies for all photocatalysts have been determined, and the effectiveness of band gap energies on polymeric composites in photocatalytic interaction has been demonstrated. From the results obtained, it was determined that the band gap energy of polymeric nanocomposites decreased compared to pure nanometal oxide, thus increasing the photocatalytic reaction rate by facilitating electron transfer. From the data obtained in the reaction kinetic studies, it was determined that each polymeric composite was compatible with the pseudo-second-order reaction kinetics for the decomposition reaction of naproxen. It is concluded that the synthesized polymeric nanocomposites have the potential to be used as a photocatalyst, removing impurities, considering the ease of production and cost.

## Figures and Tables

**Table 1:** FT-IR spectrum data of synthesized polymeric nanocomposites.

Photocatalysts	N-H tension mode (cm^−1^ )	C=C tension mode of the quinoid ring (cm^−1^ )	C=C stretching vibration mode of the benzenoid ring (cm^−1^ )	C-N tensile vibration mode (cm^−1^ )	C-H in-plane deformation	C-H out-of-plane deformation	Characteristic peaks of nanoparticles
PANI	3264	1553	1449	1282	1121	817	-
PANI/Fe_2_O_3_	3330	1577	1500	1291	1130	810	447.87, 546.6
PANI/NiO	3128	1571	1492	1281	1092	803	600, 800
PANI/SnO_2_	3259	1400	1625	1278	1107	806	667, 559, 437
PANI/WO_3_	3268	1452	1450	1280	1187	807	792, 807
PANI/ZrO_2_	3446	1550	1457	1280	1120	815	500, 600

**Table 2:** Pseudosecond-order reaction rate parameters applied for the photocatalytic and adsorption removal reaction rate of naproxen under UV-A irradiation and in the absence of light (adsorption).

Pseudosecond-degree kinetic model parameters								
Under UV degradation					Adsorption			
Name of adsorbent	q_e_, mg.g^−1^	k_2_, min^−1^	h, mg g^−1^ min^−1^	R^2^	q_e_, mg^−1^	k_2_, min^−1^	h, mg g^−1^ min^−1^	R^2^
PANI	7.96	0.030	0.52	0.9075	42.55	0.006	0.092	0.9946
PANI/Fe_2_O_3_	42.04	0.032	11.70	0.9974	39.84	0.018	12.13	0.9964
PANI/NiO	50.25	0.070	11.03	0.9991	49.50	0.033	8.13	0.9965
PANI/SnO_2_	45.24	0.041	24.15	0.9992	40.81	0.028	16.51	0.9982
PANI/WO_3_	46.72	0.043	17.88	0.9993	45.91	0.030	14.66	0.9975
PANI/ZrO_2_	45.19	0.027	23.55	0.9997	39.37	0.016	52.63	0.9993
